# Risk factors for delayed encephalopathy following carbon monoxide poisoning: Importance of the period of inability to walk in the acute stage

**DOI:** 10.1371/journal.pone.0249395

**Published:** 2021-03-31

**Authors:** Yasuhiro Suzuki

**Affiliations:** Department of Neurology, Shizuoka Saiseikai General Hospital, Shizuoka, Japan; Universidade Federal de Minas Gerais, BRAZIL

## Abstract

**Objective:**

Delayed neurological sequelae (DNS) is a serious complication that occurs after acute carbon monoxide (CO) intoxication. The study identified factors for predicting DNS development for the purpose of improving CO intoxication treatment strategies.

**Methods:**

The medical records of 65 patients admitted to Shizuoka Saiseikai General Hospital between 2004 and 2020 due to CO poisoning were retrospectively reviewed. Univariate and multivariate logistic regression analyses were performed, using a range of evaluated items as explanatory variables and the development of DNS as the response variable.

**Results:**

Patients who developed DNS were found to have higher peak creatine kinase (CK) (odds ratio, 1.0003; 95% CI, 1.0001–1.0005; P<0.001), and experienced a greater number of days during which walking was impossible in the acute stage following intoxication (odds ratio, 1.011; 95% CI, 1.005–1.018; P<0.001) according to the univariate analysis. Multivariate analyses indicated that DNS development was related to the score, peak CK (U/L) + 40 × the number of days in which walking was impossible. The model demonstrated an area under the receiver operating characteristic curve (AUC) of 0.96 (95% CI, 0.91–1.00), and DNS was predicted with 100% sensitivity and 82% specificity.

**Conclusion:**

An indicator that incorporates the number of days that walking is impossible for a patient could be useful in planning therapeutic strategies.

## Introduction

Delayed Neurological Sequelae (DNS) is a serious complication that occurs within several days to 6 weeks after a complete or partial recovery from carbon monoxide (CO) intoxication [1–3]. The development of DNS is presumed in about 10%–20% of patients who suffer acute CO poisoning. However, the mechanisms involved are not yet well understood. Identifying patients with acute CO poisoning who are likely to develop DNS would facilitate better decision-making about treatment strategies, although achieving such an identification is itself a challenge.

Initial conscious disturbance is a known risk factor of DNS development [1,2,4]. However, Kim *et al*. [5] reported that the Glasgow Coma Scale had 80% sensitivity and 77% specificity, which renders it unsuitable as a predictor in this case. Currently there are only a small number of parameters that are deemed useful in predicting DNS [6–8]. It may be possible to evaluate the severity of a patient’s acute symptom up to the point at which the symptoms disappear. The purpose of this study was to investigate associations between the duration of acute symptoms and the development of DNS among patients who suffered acute CO poisoning.

## Methods

### Study design and patients

The study involved patients with CO poisoning hospitalized in Shizuoka Saiseikai General Hospital between 2004 and 2020. A total of 158 patients were diagnosed with acute CO poisoning during this period. The diagnosis of CO poisoning was confirmed by patient medical history. Medical records of confirmed patients were then retrospectively analyzed. Of the patients so diagnosed, 93 were excluded for the following reasons.

1. Patient subsequently suffered a cardiac arrest (four patients).
2. Patient did not recovered consciousness or was unable to communicate verbally in the acute stage following intoxication (three patients).
3. Patient failed to attend follow-up at 6 weeks after the initial CO exposure (86 patients).

After the exclusion of non-eligible patients, the remaining 65 patients formed the study sample. This study was conducted according to the Declaration of Helsinki and was reviewed and approved by the Ethics Committee of Shizuoka Saiseikai General Hospital. The need for written informed consent was waived because of the retrospective nature of the study.

### Grouping

The patients were classified into a DNS group (n = 15) and a non-DNS group (n = 50). DNS was defined as any neurological signs developing within 6 weeks [1,3,4] of CO intoxication. Signs included motor deficits, cognitive decline, dysphagia, extrapyramidal symptoms and incontinence. Follow-up appointments were conducted in hospital or outpatient clinics. Not all patients underwent regular examination using a standardized cognitive test. Detection of abnormalities frequently depended on subjective reporting by patients or a patient’s family members, and these were subsequently confirmed by the medical institution. Because inclusion in the DNS group was based on the detection of objective neurological signs, patients who reported only subjective symptoms including concentration impairment, emotional instability, depression, or a headache were not included in the DNS group. The DNS group included patients who showed delayed deterioration after incomplete recovery from their initial injuries.

### Medical management

Each patient received 100% oxygen by facial mask or mechanical ventilator following endotracheal intubation. Fifteen patients underwent endotracheal intubation. Hyperbaric oxygen (HBO) therapy was administered to 61 of the 65 patients. HBO therapy was applied in a multi-person chamber, administered at 2.0–3.0 atmospheres absolute (ATA) pressure for 90–120 minutes, once or twice daily. The total number of HBO sessions administered was chosen by the attending physician. The most frequently used protocol comprised a target pressure of 3.0 ATA for the first session and 2.0 ATA for remaining 19 sessions, giving a total 20 sessions over 10 days. The protocol was administered to a total of 29 patients. Three grams or more of methylprednisolone was given to four patients as corticosteroid therapy.

### Data collection

The following information was retrospectively extracted from medical records: demographic data, level of consciousness on arrival (Glasgow Coma Scale), blood pressure, voluntary or accidental exposure, concomitant use of tranquilizers, time interval from CO exposure to hospitalization, laboratory results, APACHE II score, MRI findings, the number of HBO sessions patient received, the time interval between CO exposure and receipt of HBO, and the amount of methylprednisolone given.

The extent to which day clinical manifestations normalized was recorded. The following manifestations were observed: the duration of endotracheal intubation, the duration of time before the Glasgow Coma Scale returned to normal, the point at which the patient became able to speak their own name, the point at which the patient became able to orally eat a meal of over 1000 kcal, and the point at which the patient became able to walk five meters or more without assistance. In some cases, the patient did not recover completely. For example, seven patients were unable to walk throughout the observation period (median, 90; range, 42–314 days). In such cases, a recovery period of 365 days was recorded for the purpose of the logistic regression analysis.

Peak (the most extreme) values of the laboratory data throughout the hospitalization period were often different from initial laboratory data; in such cases both values were registered. Creatine kinase (CK) and lactate dehydrogenase (LDH) activity were measured by a spectrophotometric method, standardized by the Japan Society of Clinical Chemistry. The normal range was defined as 41–153 U/L for CK and 124–222 U/L for LDH.

Eight patients developed DNS during initial hospitalization prior to recovering completely. The number of HBO sessions recorded for such patients was counted as the number of sessions prior to DNS occurrence. MRI results were interpreted by a radiologist. In cases in which a radiologist report was not available, images were interpreted by the researcher.

### Statistical analysis

We compared each variable according to the presence or absence of DNS. Fisher exact test was performed for categorical variables, and two-tailed Welch t-test was performed for continuous variables.

Logistic univariate and multivariate regression analyses were performed using evaluated items as explanatory variables and DNS development as the response variable. For variable selection, all possible models were tested using a forward selection procedure. An R script written by Aoki Shigenobu (http://aoki2.si.gunma-u.ac.jp/R/all.logistic.html) was used to select the logistic model with the highest likelihood ratio test (LRT) statistic from the candidate models. The significance of the model was tested using LRT and Wald test. For comparison of goodness-of-fit among the models, the LRT statistic and Akaike information criterion were used.

Receiver operating characteristic (ROC) curves were constructed to establish the cutoff points for variables that optimized sensitivity and specificity in predicting DNS development via Youden Index. A P-value < 0.05 was considered significant. All statistical analyses were performed using R software for Windows, version 3.6.3.

## Results

### Baseline demographic and clinical characteristics

A total of 65 patients with acute CO poisoning were included in the analysis. Of these, 45 (69%) were men and the median age was 46 years (interquartile range, 35–61 years).

In approximately half of all cases, the most extreme points recorded in the laboratory data did not coincide with the initial values recorded. For example, the initial creatine kinase (CK) value and peak CK value were different in 22 cases. In the majority of such cases, peak values were recorded on the following day (14 cases). Initial lactate dehydrogenase (LDH) values and peak LDH values were different in 17 cases. The peak was recorded on the following day in five cases.

Fifteen patients (23%) developed DNS, with a median interval from CO exposure of 25 days (range, 11–41 days; interquartile range, 16–30 days). Six patients showed complete recovery in the acute stage, whereas nine patients showed incomplete recovery before developing DNS. Symptoms of DNS included disorientation (15 patients), difficulty in standing and walking (14 patients), rigidity (9 patients), mutism (7 patients), dysphasia requiring tube feeding (5 patients), and tremor (3 patients).

Table 1 shows the baseline characteristics of DNS and non-DNS groups including demographic data, clinical results and laboratory findings for all 63 patients. There were no significant differences between groups for the following items: sex, age, carboxyhemoglobin, base excess, anion gap, creatinine, time to hospitalization, time to hyperbaric oxygen (HBO) treatment, number of HBO sessions, endotracheal intubation period, amount of corticosteroid administered, length of time for which the patient was unable to eat, or time until normality according to the Glasgow Coma Scale.

**Table 1:** Baseline characteristics of participants with and without delayed neurological sequelae (DNS).

Characteristics	Total (n = 65)	DNS group (n = 15)	Non-DNS Group (n = 50)	P value
Male	45 (69%)	9 (60%)	36 (72%)	0.5
Age, years	46 (35–61)	44 (36–53.5)	46 (35.25–61)	0.5
Attempted to commit suicide	41 (63%)	15 (100%)	26 (52%)	<0.001
Concomitant use of tranquilizers	13 (20%)	6 (40%)	7 (14%)	0.06
Time from CO exposure to visiting hospital, hours	1 (0.5–2)	1 (0.75–1.25)	1 (0.5–2)	0.9
Glasgow Coma Scale on arrival	11 (5–15)	5 (4–6.5)	14 (7–15)	0.0002
Systolic blood pressure on arrival, mmHg	120 (104–141)	111 (94.5–116.5)	125 (110–145.2)	0.007
APACHE II score on arrival	11 (6–18)	18 (13–20.5)	9 (6–15)	0.006
Carboxyhemoglobin on arrival, percent	21 (14.5–31.4)	18 (8.05–26.95)	21.25 (15.32–32.6)	0.2
Base excess on arrival, mmol/L	-1.8 (-4–0.4)	-3.3 (-5.1 –-1.85)	-1.3 (-3.2–0.95)	0.3
Anion gap on arrival, mmol/L	13 (9–16)	14 (10.7–16)	12 (9–15.75)	0.8
CK on arrival, U/L	203 (102–1538)	3156 (1340–7478)	146.5 (88–425.5)	0.009
LDH on arrival, U/L	240 (188–325)	400 (234–557)	230.5 (184.5–296)	0.02
Peak white blood cell count, 10^3^/micro L	12500 (8710–17700)	18110 (14250–19865)	10390 (7650–14540)	<0.001
Peak CK, U/L	382 (111–2306)	4860 (1731–10194)	176 (98–534)	0.03
Peak LDH, U/L	266 (194–400)	506 (294.5–883)	235.5 (188–301.2)	0.003
Peak blood urea nitrogen, mg/dl	19 (15–24)	24 (19.5–42.25)	17 (14.25–23)	0.01
Peak creatinine, mg/dl	0.8 (0.62–1.03)	1.09 (0.825–1.61)	0.795 (0.6–0.985)	0.03
Peak C-reactive protein, mg/dl	1.35 (0.2–7.6)	8.1 (1.475–15.75)	0.55 (0.155–3.873)	0.02
Endotracheal intubation	15 (23%)	6 (40%)	9 (18%)	0.09
Endotracheal intubation period, days	0 (0–0)	0 (0–2)	0 (0–0)	0.6
Recovery period to GCS 15, days	1 (0–5)	7 (4–17)	0.5 (0–1.75)	0.1
Period patient was unable to speak their name, days	0.2 (0–2)	2 (1–3.5)	0 (0–0.9)	0.02
Period oral eating was impossible, days	1 (0.5–4)	5 (2.5–7)	1 (0–1.75)	0.3
Period walking was impossible, days	2 (1–10)	120 (10–365)	1.5 (0.125–4.5)	0.003
Interval from CO exposure to HBO treatment, hours	5 (4–10) (n = 61)	6 (5–8) (n = 13)	5 (3–11) (n = 48)	0.4
Number of HBO sessions	20 (7–20)	20 (9.5–22)	20 (7–20)	0.67
All acute brain lesion on DWI	22 (42%) (n = 52)	11 (79%) (n = 14)	11 (29%) (n = 38)	0.003
Acute brain lesion in globus pallidus	20 (38%)	9 (64%)	11 (29%)	0.02
Acute brain lesion in other white matter	9 (17%)	7 (50%)	2 (5%)	<0.001

Continuous data are expressed as medians (interquartile range), categorical data as frequencies (proportion).

Statistical comparisons were made using Welch t-test or Fisher exact test.

Abbreviations: DNS, delayed neurological sequelae; CO, carbon monoxide; GCS, Glasgow Coma Scale; CK, creatine kinase; LDH, lactate dehydrogenase; HBO, hyperbaric oxygen; DWI, diffusion-weighted magnetic resonance imaging.

### Univariate logistic analysis

Table 2 contains variables that were found to be significant and returned a large likelihood ratio test (LRT) statistic for DNS occurrence in the univariate logistic regression analysis. The LRT statistic was calculated by comparison to a null model. The area under the receiver operating characteristic curve (AUC), cutoff point, sensitivity, and specificity are also shown. The three items with the strongest relationship with DNS development were peak LDH value, peak CK value, and the time for which walking was impossible. The AUCs for these items were approximately 0.9. For the variables CK, LDH, white blood cell count, blood urea nitrogen, creatinine, and C-reactive protein, a larger LRT statistic resulted when peak values were used compared with when initial values were used.

**Table 2:** Factors strongly associated with DNS development by univariate logistic regression analysis.

Variable	LRT statistic	AIC	Odds ratio (95% CI)	P value (LRT)	AUC (95% CI)	Cutoff value	Sensitivity	Specificity
Period walking was impossible, days	19.9	54.4	1.011 (1.005–1.018)	<0.001	0.91 (0.84–0.99)	8.5	0.87	0.84
Peak LDH, U/L	19.3	55.0	1.006 (1.003–1.010)	<0.001	0.84 (0.73–0.95)	393.5	0.67	0.86
Peak CK, U/L	18.1	56.2	1.0003 (1.0001–1.0005)	<0.001	0.90 (0.83–0.97)	547.5	1	0.76
Attempted to commit suicide	16.4	57.9	–––^a^	<0.001	0.74 (0.67–0.81)	0.5	1	0.48
CK on arrival, U/L	15.9	58.3	1.0004 (1.0001–1.0006)	<0.001	0.88 (0.78–0.98)	590	0.93	0.82
Acute lesion on DWI (n = 52)^b^	10.8	54.0	9.00 (2.04–39.8)	0.001	0.75 (0.61–0.88)	0.5	0.79	0.71

^a^Odds ratio for suicide attempts could not be estimated because of complete separation (all DNS patients had attempted suicide).

^b^The number of patients who underwent MRI was 52; therefore, the statistic cannot be directly compared with other items.

Area under the receiver operating characteristic curve (AUC), cutoff values, sensitivity, and specificity for predicting DNS development are added.

Abbreviations: DNS, delayed neurological sequelae; LRT statistic, likelihood ratio test statistic compared with a null model; AIC, Akaike information criterion; OR, odds ratio; CI, confidence interval; AUC, area under the receiver operating characteristic curve; LDH, lactate dehydrogenase; CK, creatine kinase; GCS, Glasgow Coma Scale; DWI, diffusion-weighted magnetic resonance imaging.

MRI was evaluated in only 52 patients. Consequently, the relevance of MRI abnormality between the DNS and non-DNS groups could not be directly compared with that of the other variables, evaluated in 65 patients. Comparisons made among the 52 patients showed that the LRT statistic of acute brain lesion on MRI was the sixth largest, smaller than that of CK value on arrival.

### Multivariate analysis

Table 3 shows the results of three multivariate models. Among the bivariate models, the LRT statistic and AUC were largest for Model 1. The explanatory variables included in Model 1 were peak CK value and the time for which walking was impossible. The next largest LRT statistic was that obtained in Model 2. The explanatory variables of Model 2 were peak LDH value and the time for which walking was impossible.

**Table 3:** Multivariate logistic regression analysis for delayed neuropsychological sequelae.

	**Model 1**		**Model 2**		**Model 3**	
	Adjusted OR (95% CI)	P value (Wald)	Adjusted OR (95% CI)	P value (Wald)	Adjusted OR (95% CI)	P value (Wald)
Period walking was impossible, day	1.012 (1.005–1.019)	0.001	1.011 (1.005–1.018)	0.001	1.016 (1.006–1.025)	0.002
Peak creatine kinase, U/L	1.0003 (1.0001–1.0005)	0.004			1.0004 (1.0001–1.0007)	0.003
Peak lactate dehydrogenase, U/L			1.007 (1.002–1.011)	0.003		
Number of HBO sessions					0.92 (0.82–1.03)	0.12
LRT statistic	37.18		35.11		41.77	
AIC	39.05		41.12		36.46	
AUC (95% CI)	0.96 (0.91–1)		0.93 (0.86–1)		0.95 (0.88–1)	
sensitivity	1		0.93		0.93	
specificity	0.82		0.86		0.92	

Abbreviations: OR, odds ratio; CI, confidence interval; CK, creatine kinase; LDH, lactate dehydrogenase; HBO, hyperbaric oxygen; LRT statistic, likelihood ratio test statistic compared with a null model; AIC, Akaike information criterion; AUC, area under the receiver operating characteristic curve.

**Fig 1:**
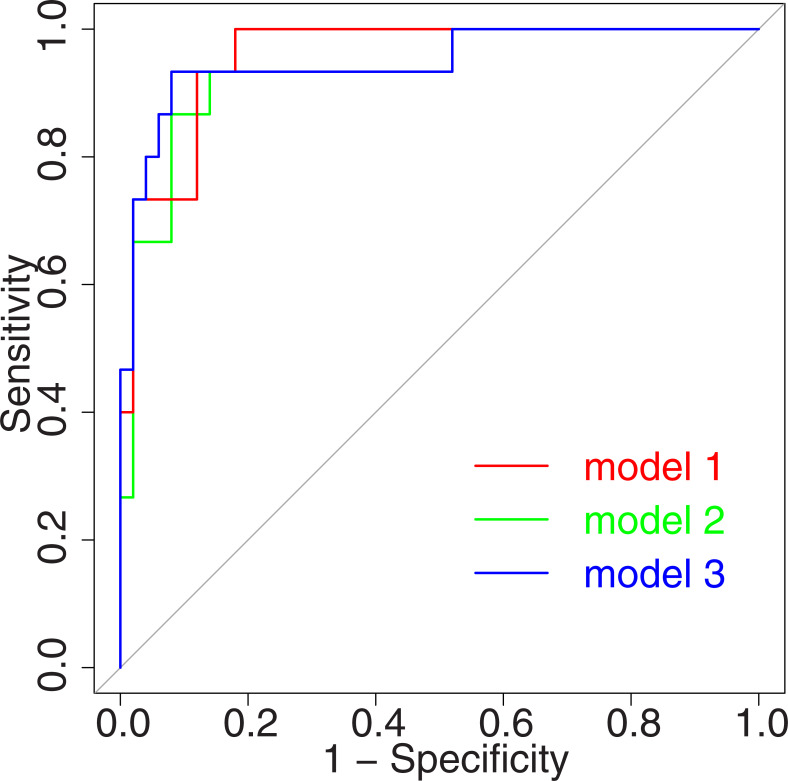
Receiver operating characteristic curves of three models for discriminating delayed neurological sequelae.

Model 3 was constructed by adding the number of HBO sessions as an explanatory variable to Model 1. Among the trivariate models in the study, Model 3 returned the largest LRT statistic. The number of HBO sessions in Model 3 was found to be non-significant by a Wald test. However, the number of HBO sessions in Model 3 was found to be significant using a LRT compared with Model 1 (LRT statistic = 4.59, df = 1, P = 0.03). When the presence of an MRI abnormality was included as an explanatory variable in the multivariate analysis of the 52 patients receiving MRI, the models with the largest LRT statistic were the same as those calculated from all 65 patients. Fig 1 shows the ROC curves for Models 1, 2, and 3. The ROC curves each display AUCs above 0.9.

**Fig 2:**
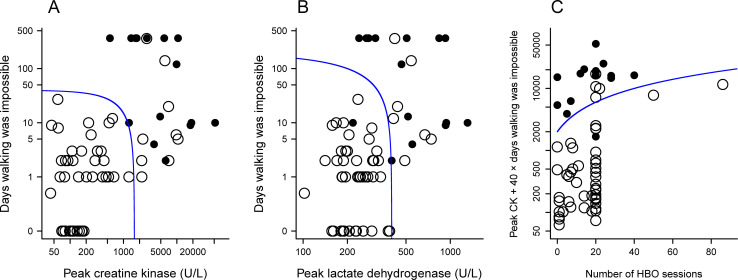
Relationship between explanatory variables and the development of delayed neurological sequelae (DNS). Closed circles indicate DNS patients and open circles indicate non-DNS patients. A. The explanatory variables are peak creatine kinase (peak CK, x-axis) and the time for which walking was impossible (WALK, y-axis). The blue line indicates the equation, peak CK (U/L) + 40 × WALK (days) = 1600. B. The explanatory variables are peak lactate dehydrogenase (peak LDH, x-axis) and the time for which walking was impossible (WALK, y-axis). The blue line indicates the equation, peak LDH (U/L) + 2 × WALK (days) = 400. C. The explanatory variables are peak CK, WALK and the number of hyperbaric oxygen (HBO) sessions. On the y-axis is the score, peak CK (U/L) + 40 × WALK (days). The blue line indicates the equation, peak CK (U/L) + 40 × WALK (days) - 200 × the number of HBO sessions = 2000.

For the optimal cutoff points, see Fig 2.

Fig 2 shows the relationship between DNS occurrence and the explanatory values in the three models. The threshold lines were determined using simplified logistic regression coefficients. For example, the regression equation of model 1 was logit (P) = −3.29 + 0.00031 × peak CK (U/L) + 0.012 × WALK (days), where WALK denotes the time for which walking was impossible during the acute stage of intoxication. By assigning zero to logit (P), the following approximate equation of the threshold line in Fig 2A was obtained: ‘peak CK (U/L) + 40 × WALK (days) = 1600’. The cutoff point is visualized at 100% (15/15) sensitivity and 82% (41/50) specificity in Fig 2A.

Fig 2C depicts the trivariate Model 3. The score, peak CK (U/L) + 40 × WALK (days), which was obtained above, is measured on the y-axis. Ten severe patients with scores above 12000 developed DNS despite treatment with 20 or more sessions of HBO therapy. DNS was rarely observed if the scores were below 2000. Of moderate patients with scores between 2000 and 12000, three patients who received fewer than 10 sessions of HBO developed DNS, while eight patients who received 20 sessions of HBO did not.

## Discussion

The study results show that the number of days in which walking was impossible during the acute stage (WALK) is a predictor for the occurrence of DNS with high sensitivity (87%) and high specificity (84%) at a cutoff point of 8.5 days. Results confirm the usefulness of laboratory data such as CK [4,8–10], LDH [11], and WBC [2,4,6], and show that peak values are generally more useful than initial values for the purpose of prediction.

Moreover, we demonstrate that the score, peak CK (U/L) + 40 × WALK (days), is a good screening tool for predicting DNS with 100% sensitivity and 82% specificity. The usefulness of the score is comparable with the most favorable indicators reported previously [7,8]. In addition, as a practical marker it is both cost-effective and highly accessible for general medical facilities; the findings would contribute to therapeutic strategy for CO poisoning. However, at the beginning of hospitalization, physicians do not know when the patient will be able to walk. As shown in Fig 2A, patients who are able to walk within 1 day rarely develop DNS. Apart from these cases, the utility for treatment planning is limited at the time of admission.

Aside from WALK and the peak CK value, based on Model 3 the number of HBO sessions may also be involved in DNS development. The efficacy of >3 HBO sessions [12] in preventing DNS remains unconfirmed [13]. Fig 2C is based on Model 3 and suggests that DNS be prevented by performing approximately 20 HBO sessions in moderate patients (peak CK value + 40 × WALK = 2000 − 12000). However, because of the small sample size, the potential for overfitting of the data and over-interpreting the results should be considered [14]. Furthermore, the protocol for the HBO sessions was not uniform, and the multivariate analysis findings are only preliminary.

This study has several limitations. First, the retrospective nature of the study increases the potential risk for selection bias or information bias. Second, the sample size is relatively small and is drawn from a single hospital, limiting the generalizability of the findings. Third, a symptom-based diagnostic criterion was adopted with the choice of DNS. This may introduce reporting bias into the results. Fourth, in the patient in whom the observation ended while the DNS symptoms (e.g., walking disability) continued, the symptom duration was recorded as 365 days for statistical analysis. Moreover, for the nine patients with incomplete recovery from acute poisoning, the symptom duration encompassed both the acute and delayed symptoms. However, subtraction of the delayed symptom period from the entire symptom period was impossible. They would cause information bias.

## Conclusions

The preliminary study suggests that the clinical score (peak CK (U/L) + 40 × WALK (days)) can serve as an early predictor for the development of DNS. This predictor might be useful for planning CO poisoning therapy.

## Supporting information

S1 Table Patient data. (CSV)

## Abbreviations

CO: carbon monoxide
DNS: delayed neurological sequelae
CK: creatine kinase
LDH: lactate dehydrogenase
HBO: hyperbaric oxygen
LRT: likelihood ratio test
WALK: time for which walking was impossible during the acute stage of intoxication
